# Using health information technology to support the needs of Children with Medical Complexity: Mapping review of consumer informatics applications

**DOI:** 10.3389/fdgth.2022.992838

**Published:** 2022-12-23

**Authors:** Onur Asan, Safa Elkefi, Katharine N. Clouser, Stephen Percy

**Affiliations:** ^1^ School of Systems and Enterprises, Stevens Institute of Technology, Hoboken, NJ, United States; ^2^ Department of Pediatrics, Hackensack University Medical Center (HUMC), Hackensack, NJ, United States

**Keywords:** children with medical complexity, caregivers, consumer informatics, telehealth, mobile health, telemedicine, patient portal, technology

## Abstract

**Background:**

Children with medical complexity (CMC) are fragile populations that require continuous care and supervision. CMC family caregivers experience many challenges trying to address CMC patients' needs which puts these caregivers in a stressful situation that may negatively impact the care of CMC patients. Consumer informatics might help these caregivers in coordinating care. However, few consumer informatics applications explicitly focus on supporting CMC caregivers' needs.

**Objective:**

This systematic mapping literature review aims to provide an overview and a structured understanding of the consumer informatics designed for CMC and their caregivers.

**Methods:**

We followed a systematic mapping literature review process to provide an overview of the existing Consumer Informatics literature for CMC, which is the scope of our study. We screened IEEE Xplore, Web of Science, and PubMed databases using a preset list of mesh terms that cover the use of medical informatics by children with medical complexities and their caregivers. The selected articles are peer-reviewed English publications that were empirically validated from January 2002 to January 2022. After selecting and filtering the articles, we analyzed them based on the preset mapping questions using the following criteria: publication year, publication source, research type, contribution type, empirical type, the need addressed, target audience, technology users, and consumer informatics' type.

**Results:**

The initial search resulted in a number of (*N* = 2,275) articles, and 17 selected publications were included. The results showed an increasing interest in CMC consumer informatics publications over time. Most of the studies were published in 2021, and feasibility research is the dominant research type. The most used technology was telehealth and telemedicine, followed by mobile health. The technologies addressed various needs, including; coordination & follow-up, medical safety, education & social support, daily living activities, shared decision making, information seeking, and emotional support. Most of the efforts were focused on ensuring good coordination and follow-up.

**Conclusions:**

CMC consumer informatics is a promising research field to present novel initiatives and approaches to manage the caregivers' workload. Future research should be shifted toward providing more evidence-based studies to examine the effectiveness of CMC consumer informatics solutions and identify the related challenges and limitations.

## Introduction

Despite significant attention being given to children with medical complexity (CMC) in clinical settings, a lack of consistency exists in how these children are described and defined in the literature (1). Specifically, Dewan et al. defined the CMC based on the presence of several complex chronic conditions, which are often severe, functional limitations that are significant and heavily reliant upon technology, and the high utilization of health care (2). According to Berry et al., CMC refers to a subcategory of children with special needs: a group of children with chronic or complex medical conditions often associated with medical fragility (3). CMC is a small (accounting for <5% of the overall pediatric population) but a growing patient population with widely varied needs (4). Even though CMC represents a small portion of the pediatric population, they face the same healthcare challenges as other children, such as high healthcare costs, unmet healthcare needs, poor quality treatment, and no effective treatments (1).

Considering the complexity of their medical conditions, these children frequently need access to healthcare services, continuous home care, education, and continuous support from family members (1). Moreover, because children with significant medical complexities experience the interaction between primary and co-morbid diagnoses, they have extreme functional limitations and have continuous access to health services with high rates of acute, rehabilitation, and community care (5). Families of CMC who live far from large urban centers and specialized clinics may have difficulty receiving medical treatment due to travel costs (6). Providing quality care at home increases the value of healthcare by avoiding costly hospital settings and reducing overall healthcare costs (7). However, caring for children with chronic or complex medical needs requires extraordinary sacrifices for caregivers, including parents and other family members. It involves caregivers taking on additional duties and acting on multiple roles (8). These numerous responsibilities often place caregivers at risk of stress or burnout (9).

Existing care delivery models offer limited support to CMC and families. Conventional health care systems are not designed to meet the unique needs of CMC and their families, with >95% lacking specific programs for them (10). The nature of these models creates many problems for care coordination for CMC. As central figures in CMC overall care, caregivers (parents) can act as “safety nets” for their children and conduits to outpatient medical history and home care routines during inpatient admissions. They have active roles as “in-home care providers” (11). They are in situ’ experts' on their child's unique needs and often subtle responses to pain and illness. Since families spend considerable time in the care of CMC, they can assist in detecting, explaining, or correcting potential errors (12). Furthermore, the technology used for care coordination and information transfer of these patients heavily is EHR, which is generally incomplete due to the involvement of various providers across different healthcare systems. On the other hand, not much consumer informatics can help caregivers maintain the necessary information for the care of their children despite its potential benefits in pediatric care (13). In addition to providing information to patients and the public, Consumer Health Informatics facilitates self-care promotion, enables informed decision-making, encourages healthy behaviors, and facilitates peer-to-peer exchange of information (14). In this mapping review, we explore the state of the art of consumer health information technologies used by CMC patients and their families to facilitate care coordination of CMC. We also explored how these technologies impact the overall outcomes in CMC care as a part of the patient and family-centered care.

## Methods

### Study design

We performed a mapping review to explore the use of consumer informatics, specifically applications used by CMC patients and their caregivers (parents, other family members, or others) to satisfy the needs of these patients. We focused on applications that can support the following needs: information seeking, shared decision-making, daily living activities, coordination and follow-up, medical safety, emotional support or education, and social support. Our protocol was registered with the Open Science Framework on https://osf.io/kq8dm/.

Mapping reviews are well-developed approaches that cover the representative literature (not exhaustive) for exploring and demonstrating trends in a given topic and duration. We followed the mapping methodology process Paterson et al. (15) suggested. The method involves selecting relevant publications, developing a classification scheme, and mapping publications systematically. The principal objective of a systematic mapping study is to structure the research area and provide an overview of the available literature, primarily by investigating the covered topics and classifying the public contributions (16).

### Mapping questions (MQs) and data extraction strategy

The mapping review questions (MQs) were defined to provide a structured understanding and overview of CMC's existing Consumer Informatics literature in the selected databases (15). Table 1 presents the MQs of this study and their rationale. The data extraction from the selected studies focused primarily on providing answers to the MQs according to the criteria presented.

**Table 1:** Mapping questions and their rationale.

Mapping Questions	Rationale
**MQ1:** How has the frequency of publications addressing CMC consumer informatics changed over time?	Identifying the publication year and term used can assist in suggesting the publication trend.
**MQ2:** Which publication channels are the main target for CMC consumer informatics research?	Identifying the publication channel and the publication source of each study.
**MQ3:** What are the research types of studies addressing CMC consumer informatics?	Research types can be classified as: (Solutions proposal, Review, Exploratory analysis, Opinion paper, Validation Research, Evaluation Research, Feasibility Study, and Books or chapters).
**MQ4:** What are the contributions of published CMC consumer informatics studies?	The contributions can be classified as follows: (Tool-based technique, Model, Method, Guidelines, Framework, Protocol, Perspectives, Usability, and Advantages & challenges).
**MQ5:** Are CMC consumer informatics studies empirically validated or evaluated?	The empirical types can be classified as follows: (Experiment, Case study, Questionnaire, Interview, Mixed methods, focus group, other, or none).
**MQ6:** What are the needs addressed in CMC consumer informatics literature?	Identifying the need addressed in each study (based on the identified needs in the map of this study)
**MQ7:** Who are the target audience in CMC consumer informatics studies?	Identifying the targeted cohort group
**MQ8:** Who are the users that literature tried to support most?	Identifying the targeted CMC users based on specific problems (Needs, Chronic conditions, Functional limitations, Health care use).
**MQ9:** What is the type of consumer informatics used?	Identifying the type of health consumer informatics used by the study users.

### Search strategy

**Figure 1:**
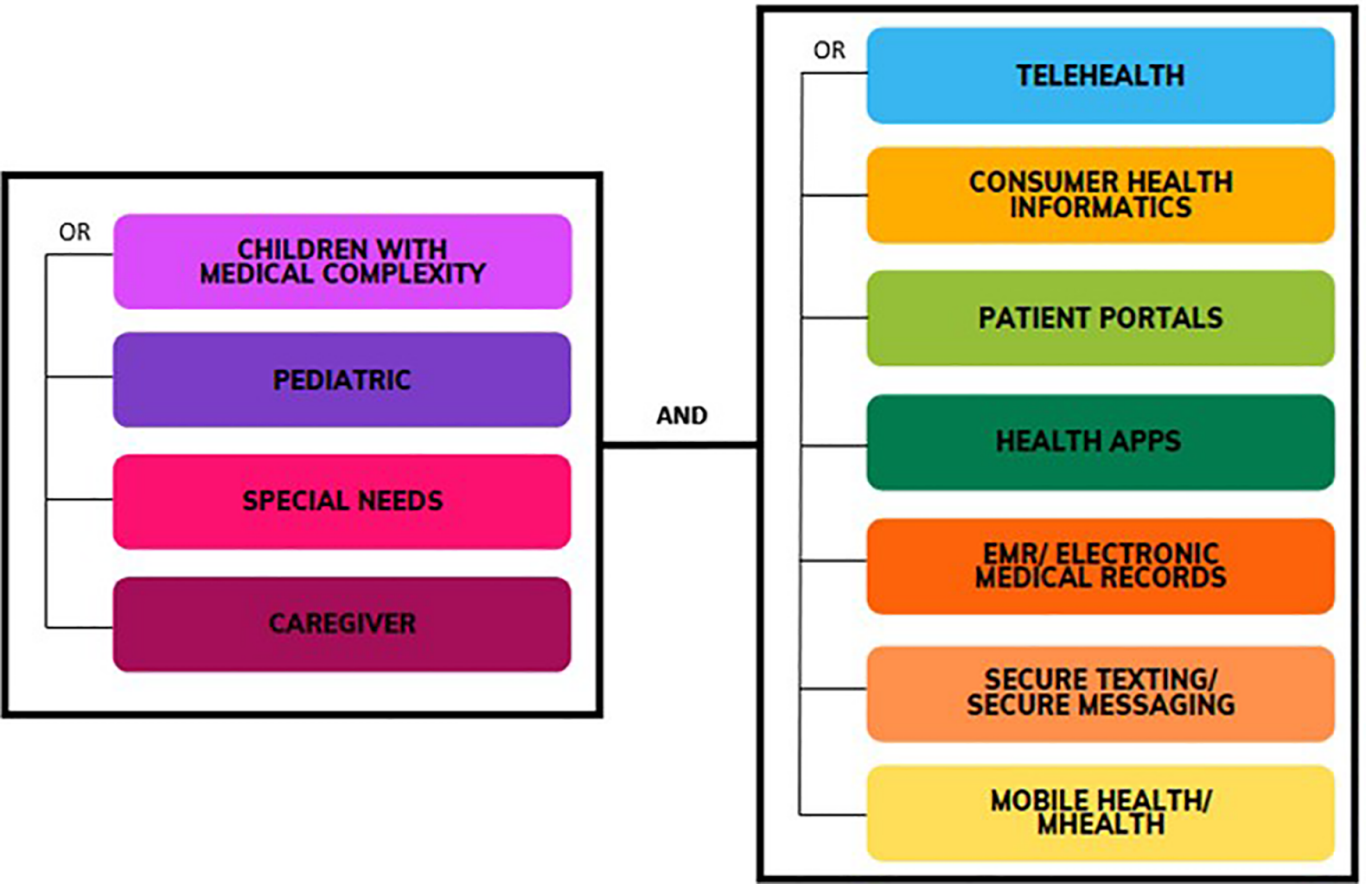
Mesh terms used in this systematic mapping review.

Three Literature databases (PubMed, Web of Science, IEEE Xplore) were searched to support the following research question: “How can the consumer informatics support the CMC and their caregivers’ experience?”. The aim of the selection process was to identify the articles that are most relevant to the objective of this mapping study. To further focus the search and include relevant studies, the search was focused on the titles of the publications. The Mesh terms presented in Figure 1 were used. The Mesh terms used are classified into two groups: first is to capture the users' groups (CMC and caregivers), and the second is to capture the health consumer informatics. For example, a search combination would be [“Children with Medical Complexity” AND (“telehealth” OR “consumer health informatics” OR “patient portals” OR “health App” OR “EMR” OR “electronic medical records” OR “Secure Texting” OR “secure messaging” OR “mobile health” OR “mhealth”)].

The search strings were formulated to include a broad selection of literature. They were not combined in one search string to identify the number of results for each term separately. The search was conducted on February 1, 2022. We covered the period from January 1, 2002, to January 31, 2022. The search yielded 2,275 results initially.

### Paper selection

A series of screening stages were carried out based on preset inclusion and exclusion criteria. The author (SE) retrieved candidate papers from the search results and entered information in an Excel (Microsoft Corporation) file that was shared with the other authors for revision. The two authors (OA, SE) examined the title, abstract, and keywords based on the inclusion and exclusion criteria and made the final decisions. We only left English peer-reviewed studies (notes, editorials, letters, and abstracts were excluded) that suggest an empirically validated technology. Only studies reporting outcomes of CMC consumer technology with explicit use of the term CMC were included. Finally, we excluded any studies that do not address consumer technology use for CMC or their family caregivers.

### Synthesis method

**Figure 2:**
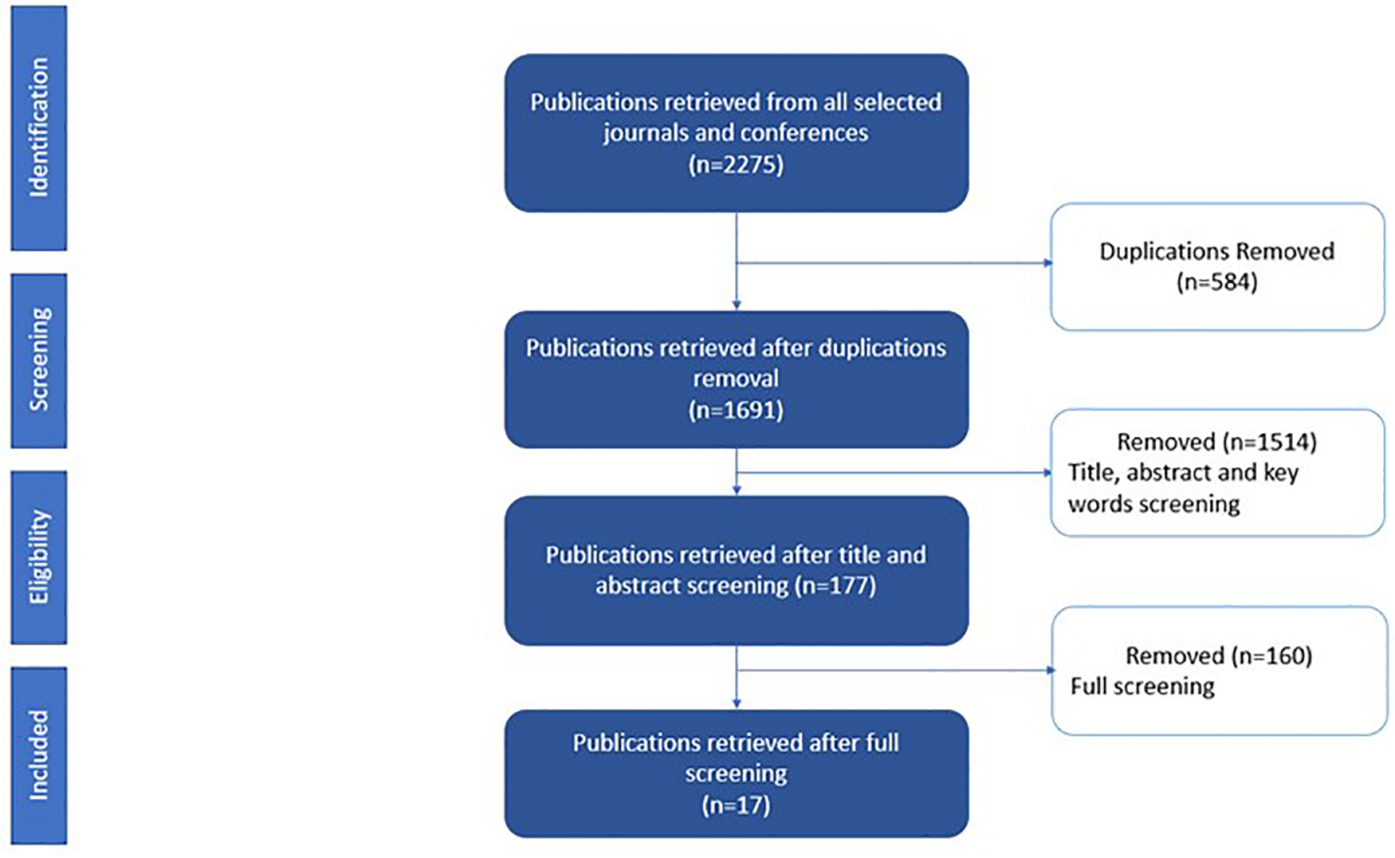
PRISMA selection process flow chart.

The synthesis method used in this study consisted of the following steps. First, we analyzed the 17 selected studies to extract information presented in the *Data Extraction Strategy* subsection. Second, we classified the studies by enumerating the number of publications per MQ. It should be noted that selected publications addressing more than one health issue (MQ6) and more than one cohort group (MQ7) were counted in each category. Third, presenting the classification results in figures and charts to visualize the results to facilitate the analysis. Last, we proposed a narrative summary to describe the principal findings of our study. Figure 2 shows the selection results. Seventeen papers (out of 177 candidate studies) were included in the final selection.

## Results

In this part, we summarize the mapping study results and the results of the MQs. Table 2 also illustrates details of the selected papers for each MQs.

**Table 2:** Key findings of the study.

N	Citation	MQ1	MQ2	MQ3	MQ4	MQ5	MQ6	MQ7	MQ8	MQ9	Findings
		Year	Publication Channel	Research Type	Type of contribution	Empirical Method	Needs addressed	Target audience	Users supported	Type of technology	
1	(17)	2020	Journal of the American Medical Informatics Association AMIA	Solution proposal	Framework	Focus Groups (*C* = 10 caregivers, *H* = 20 health care providers, *P* = 10 CMC)	Coordination & follow up	Caregivers and Designers of technology, Doctors, CMC	Caregivers, Patients	Online portals/webpage	This study identified 6 key design preferences that would support CMC and caregivers’ information needs: the ability of individual caregivers to customize their homepage based on their current content priorities and having customized homepage layouts for different provider groups, a problem-based organizational framework with individual active issue pages displaying a sick plan, pertinent history, and baseline management information, flexibility in how content within each section is structured and displayed to balance the needs of families and different provider groups.
2	(18)	2018	Journal of Medical Internet Research JMIR	Validation research	Advantages and challenges	Experiment (*C* = 7 CMC caregivers, *H* = 47 HCP, *P* = 12 CMC patients and other patients from different other age groups)	Coordination & follow up	Caregivers and Designers of technology, Doctors, CMC	Caregivers, Doctors, Patients	Online portal	The tool was perceived to help improve the workflow of information and communication between care team members (doctors, patients, caregivers)
3	(19)	2017	Journal of Pediatric Health Care	Exploratory Impact study	Advantages and challenges	Focus Groups (*C* = 163 caregivers, *P* = 163 CMC patients)	Coordination & follow up	Caregivers and Designers of technology, Doctors, CMC	Caregivers, Patients	Telehealth for CMC	This study helped quantify CMC's changes in health care utilization after an intervention with APRN-mediated telehealth care coordination within a mature health care home. The interventions in this study could help decrease the number of unplanned clinical visits and increase the number of planned clinical visits. This could lead to more effective and efficient care for the growing population of CMC.
4	(20)	2019	Journal of Clinical Pediatrics	Feasibility study	Advantages and challenges	Mixed-Method (*C* = 35 caregiver parent, *P* = 47 CMC patients) Survey and Interview	Coordination & follow up/Emotional support/Shared decision making	Caregivers and Designers of technology, Doctors, CMC	Caregivers, Patients	Mobile health	In this study, parents identified Mobile Complex Care Plans accessed through online mobile portals as an essential reference and communication tool. MCCPs for CMC in a complex care program were feasible, facilitated parental engagement, and delivered timely communication.
5	(21)	2015	Maternal Child Health Journal	Exploratory Impact study	Model suggestion	Survey (*C* = 37 caregivers, *P* = 55 patients)	Coordination & follow up	Caregivers and Designers of technology, Doctors, CMC	Caregivers, Patients	Telehealth for CMC	This study's findings suggest that in an established medical home with high levels of FCC, families of CMC have unmet needs for care coordination help that the APRN telehealth care coordination model addresses.
6	(22)	2021	The Journal of Pediatrics	Feasibility study	Perspectives & Attitudes towards technology/Advantages and Challenges	Mixed-Method (*C* = 75 caregivers, *P* = 75 patients) survey and focus groups	Medical Safety/Coordination & follow up	Caregivers and Designers of technology, Doctors, CMC	Caregivers, Patients	Mobile health	This study represents an essential first step toward establishing mHealth platforms to monitor and identify health stability and instability longitudinally. Families offered several insightful improvements for future iterations of the text messaging platform.
7	(23)	2019	International Journal of Medical Informatics	Solution proposal	Perspectives & attitudes towards technology/Advantages and challenges	Focus Groups (*C* = 13 caregivers, *P* = 13 patients)	Medical Safety/Coordination & follow up/Daily living activities	Caregivers and Designers of technology, Doctors, CMC	Caregivers, Patients	Mobile health	In this study, caregivers deemed a mHealth tool beneficial and offered a set of key functionalities to meet information needs for monitoring CMC's symptoms
8	(24)	2016	Journal of Pediatric Rehabilitation Medicine	Evaluation Research	Perspectives & Attitudes towards technology	Mixed-Method (*C* = 32 caregivers, *P* = 32 patients) focus groups and surveys	Shared Decision making/Coordination & follow up	Caregivers and Designers of technology, Doctors, CMC, Policymakers, hospitals managers	Caregivers, Patients	Telehealth for CMC	Telehealth allows increased opportunities for inter-professional team members to integrate CMC's medical, rehabilitation, and education needs. Using this modality, a team caring for this complex population can work together to provide the holistic approach to care that is necessary with CMC.
9	(25)	2020	Pilot Feasibility Studies	Feasibility study	Advantages and challenges	Mixed-Method (*C* = 4 caregivers, *H* = 5 Health care providers) Interviews, surveys, and field notes	Shared Decision Making/Coordination & follow up/	Caregivers and Designers of technology, Doctors, CMC, Policymakers, hospitals managers	Caregivers, Patients	Video conference (Telemedicine)	This study proved that videoconferences were acceptable and appropriate due to benefits, including developing a shared understanding, remote physical assessment by the PCP, transparency, humanization of the care handoff, and increased PCP comfort with the care of CMC. Feasibility: Barriers included internet connection quality and scheduling constraints
10	(26)	2021	Hospital Pediatrics	Feasibility study	Perspectives & Attitudes towards technology	Mixed-Method (*C* = 62 caregivers, *P* = 62 CMC patients) Survey and Focus groups	Daily living activities/Coordination & Follow up/Emotional Support/Medical Safety/Shared Decision Making	Caregivers and Designers of technology, Doctors, CMC, Policymakers, hospitals managers	Caregivers, Patients	Mobile health	MyChildCMC subjects recorded the child's vital signs and symptoms daily for three months post-discharge and received real-time feedback. MyChildCMC was feasible and appeared effective, with the potential to detect early deteriorations in health for timely interventions that might avoid ED and hospitalizations
11	(27)	2019	Telemedicine and e-health	Feasibility study, Validation research	Usability and effectiveness	Focus Groups (*C* = 24 caregivers, *P* = 24 CMC patients)	Medical Safety/Coordination & Follow up/Information Seeking	Caregivers and Designers of technology, Doctors, CMC, Policymakers, hospitals managers	Caregivers, Patients	Telehealth for CMC	This study demonstrated the successful use of a telehealth device by a caregiver in children's homes with complex medical conditions. Caregivers were comfortable with and satisfied using the device. Clinicians found the device helpful in gathering data to inform patients’ care plans. Compared with the control group, those with access to telemedicine had fewer hospital days and reduced cost rates.
12	(28)	2018	Journal of Pediatric Health Care	Evaluation Research	Advantages and challenges	Survey (*C* = 163 caregivers, *P* = 163 CMC patients)	Daily living activities	Caregivers and Designers of technology, Doctors, CMC, Policymakers, hospitals managers	Caregivers, Patients	Telehealth for CMC	Due to challenges in the impact of telehealth on improving the patients’ and caregivers’ health-related quality of life
13	(29)	2021	Frontiers in Pediatrics	Evaluation Research	Advantages and challenges	Focus Groups (*C* = 64 caregivers, *P* = 64 CMC patients)	Daily living Activities/Coordination & Follow up/Medical Safety	Caregivers and Designers of technology, Doctors, CMC, Policymakers, hospitals managers	Caregivers, Patients	Telehealth for CMC	A telehealth-based care coordination team significantly decreased some metrics of healthcare utilization in a complex pediatric population. Future study is warranted into telemedicine for care coordination programs caring for children with medical complexity.
14	(30)	2021	Journal of Pediatric Health Care	Feasibility study, Validation research	Usability and effectiveness	Mixed-Method (*C* = 28 caregivers, *P* = 28 CMC patients) Focus groups and surveys	Coordination & follow up	Caregivers and Designers of technology, Doctors, CMC, Policymakers, hospitals managers	Caregivers, Patients	Telehealth for CMC	The application of telehealth for CMC helped in self-management and self-efficacy improvement for patients and caregivers. The application recorded high acceptability
15	(31)	2015	Journal of Pediatric Health Care	Validation research	Perspectives & Attitudes towards technology	Survey (*C* = 148 caregivers, *P* = 148 patients)	Daily Living Activities/Medical Safety/Coordination & Follow up	Caregivers and Designers of technology, Doctors, CMC, Policymakers, hospitals managers	Caregivers, Patients	Telehealth for CMC	The intervention was associated with higher ratings on measures of the child's provider, provider communication, overall health care, and care coordination adequacy, compared To controls. Higher levels of condition complexity were associated with higher ratings of overall health care in some analyses.
16	(32)	2020	Hospital Pediatrics	Solution proposal, Validation research	Model suggestion, Perspectives and challenges, usability, and effectiveness	Experiment (*C* = 14 CMC caregivers)	Emotional support/Shared decision making/Education and social support/Information seeking/Medical Safety/Daily living activities	Caregivers and Designers of technology, Doctors, CMC, Policymakers, hospitals managers	Caregivers, Doctors, Patients	Mobile health	The app's overall purpose was to centralize and coordinate all aspects of enteral tube care, such as daily routines, medications and dosages, inventory, relevant data logs, individuals involved in enteral tube care, and tube troubleshooting. Participants identified facilitating communication among different caregivers and caregiver handoffs as key to the app's overall functionality
17	(33)	2021	Journal of the American Medical Informatics Association AMIA	Solution proposal	Model suggestion	Interview (*C* = 10 CMC caregivers, *H* = 20 Doctors)	Coordination & follow up/Information Seeking	Caregivers and Designers of technology, Doctors, CMC, Policymakers, hospitals managers	Caregivers, Doctors, Patients	Patient portals	These findings provide a model for how we may leverage emerging Health Insurance Portability and Accountability Act-compliant cloud computing technologies to support families and providers in co-managing health information for CMC

**MQ1: How has the frequency of publications addressing CMC consumer technology changed over time?**

**Figure 3:**
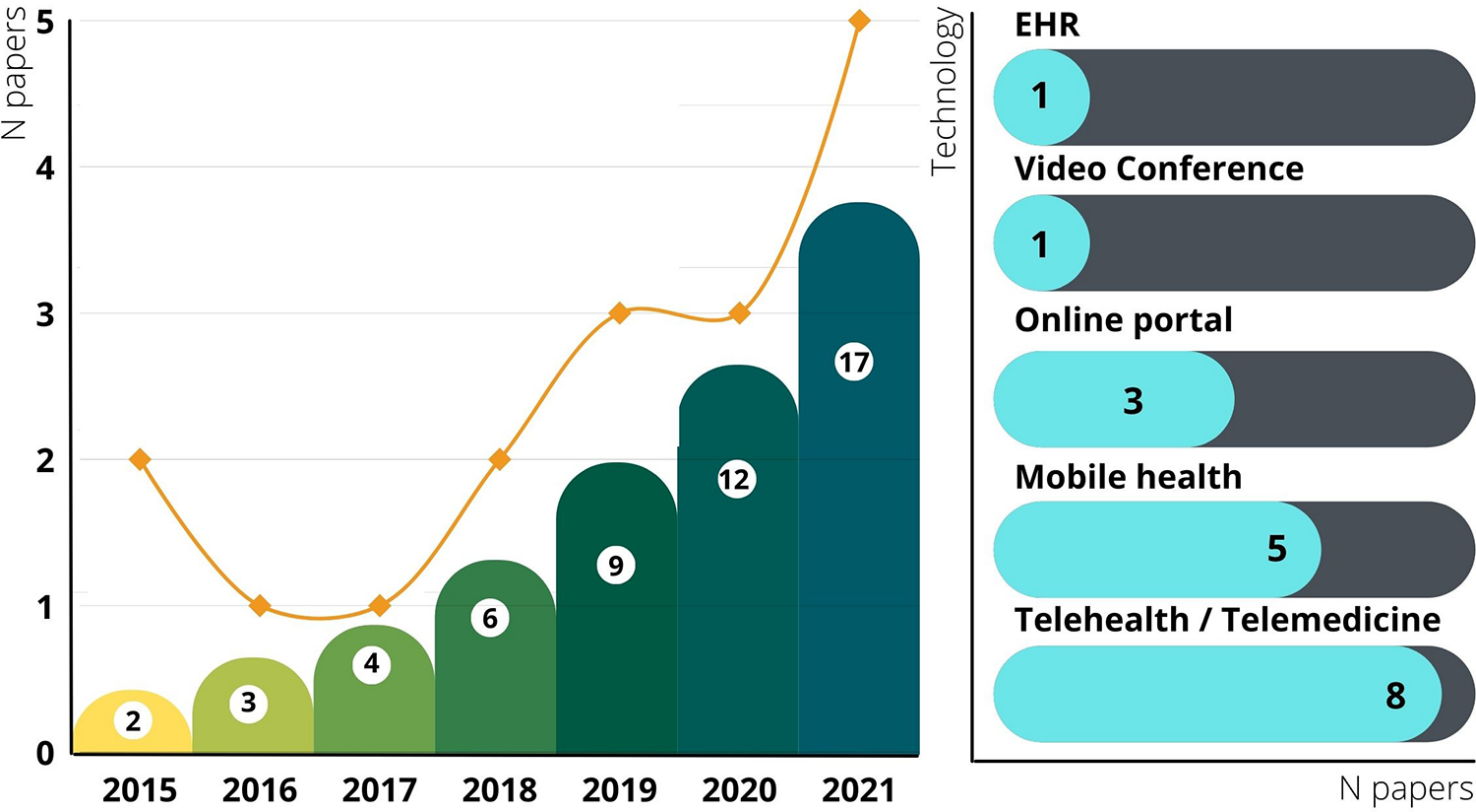
Evolution over time of the publications related to CMC and the type of technology used.

Figure 3 shows the publication trend in the selected papers. The data shows a significant increase in the number of studies covering technology that supports CMC caregiving in the past decade. We did not find any article between 2002 and 2015, which shows that this is a new gap addressed in the literature. Attention was not accorded to consumer informatics for caregivers and CMC support before 2015. The publication trend evolved from 2 articles in 2015 to 5 new articles in 2021, which correlates with the pandemic year. We estimate that the publication trend will continue to increase in the upcoming years.

**MQ2: Which publication channels are the main target for CMC consumer technology research?**

Only journal papers were included in the selected studies. The overall distribution is summarized in (Table 3).

**Table 3:** Publication channels identified in this review.

Journal	Number of Publications
Journal of the American Medical Informatics Association (JAMIA)	2
Journal of Medical Internet Research (JMIR)	1
Journal of Pediatric Health Care	4
Journal of Clinical Pediatrics	1
Maternal Child Health Journal	1
The Journal of Pediatrics	1
International Journal of Medical Informatics (IJMI)	1
Journal of Pediatric Rehabilitation Medicine	1
Pilot Feasibility Studies	1
Hospital Pediatrics	2
Telemedicine and e-health	1
Frontiers In Pediatrics	1

**MQ3: What are the research types of studies addressing CMC consumer technology?**

**Figure 4:**
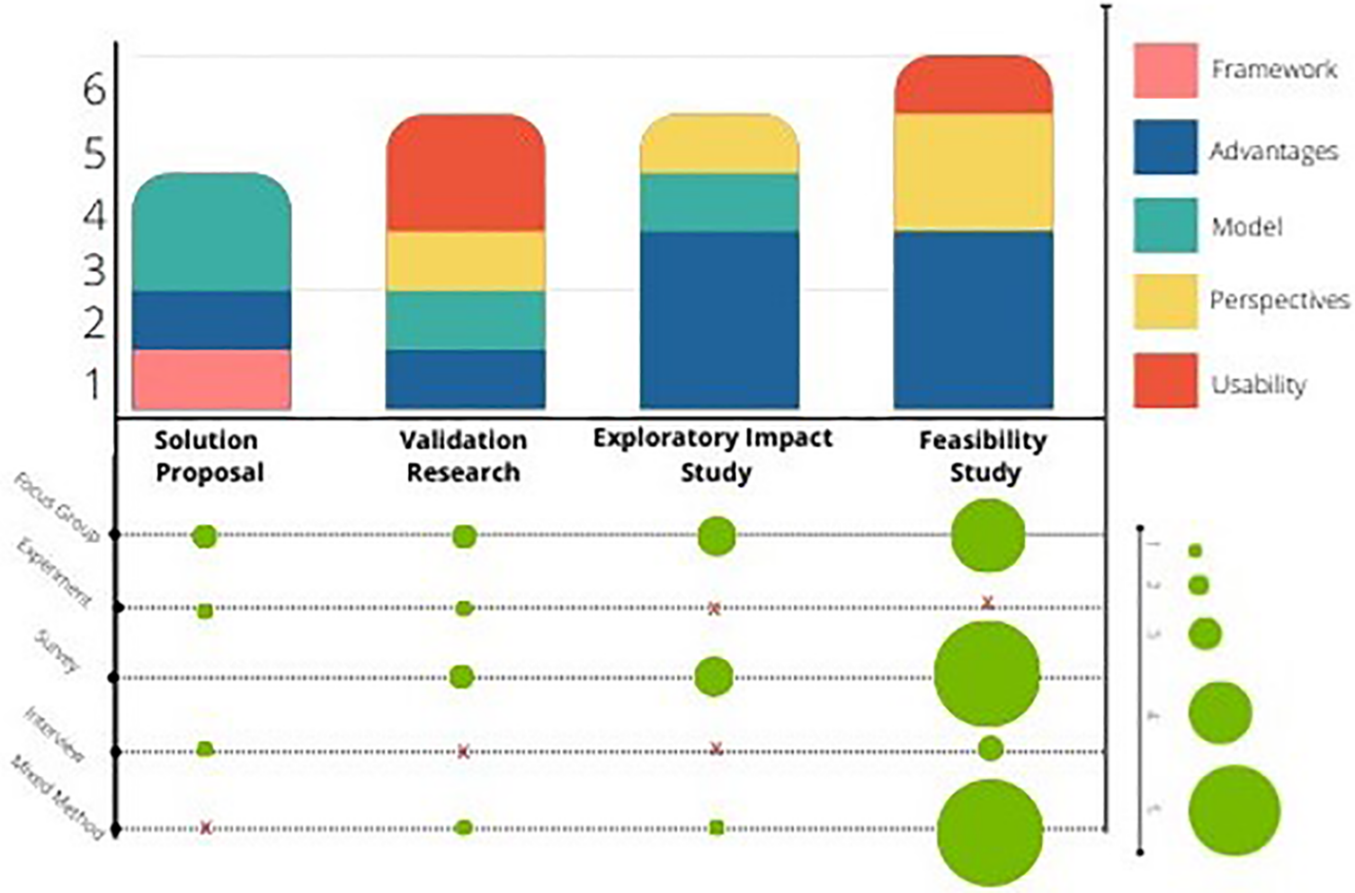
Association between research types, types of contributions, and empirical methods.

Figure 4 presents the research types identified in the selected papers. The most significant number of selected publications included feasibility studies (35.29%, *N* = 6 studies), followed by validity studies (29.41%, *N* = 5 studies), and exploratory impact studies (29.41%, *N* = 5 studies). Solution proposals consisted of 23.52% of the studies with *N* = 4.

**MQ4: What are the contributions of published CMC consumer technology studies?**

As shown in Figure 4, (47.06%, *N* = 8) of the selected studies addressed the advantages and challenges of CMC technology; 23.53% of the studies contributed with a Model Suggestion and Perspectives & Attitudes towards technology. Only one study suggested a framework.

**MQ5: Are CMC consumer technology studies empirically validated or evaluated?**

Figure 4 shows the identified empirical types of the selected papers. All the selected studies were evaluated empirically. Overall, seven studies used mixed methods. The majority of the studies used focus groups and/or surveys. Only three studies used interviews, and two used experiments.

**MQ6: What are the needs addressed in CMC consumer technology literature?**

**Figure 5:**
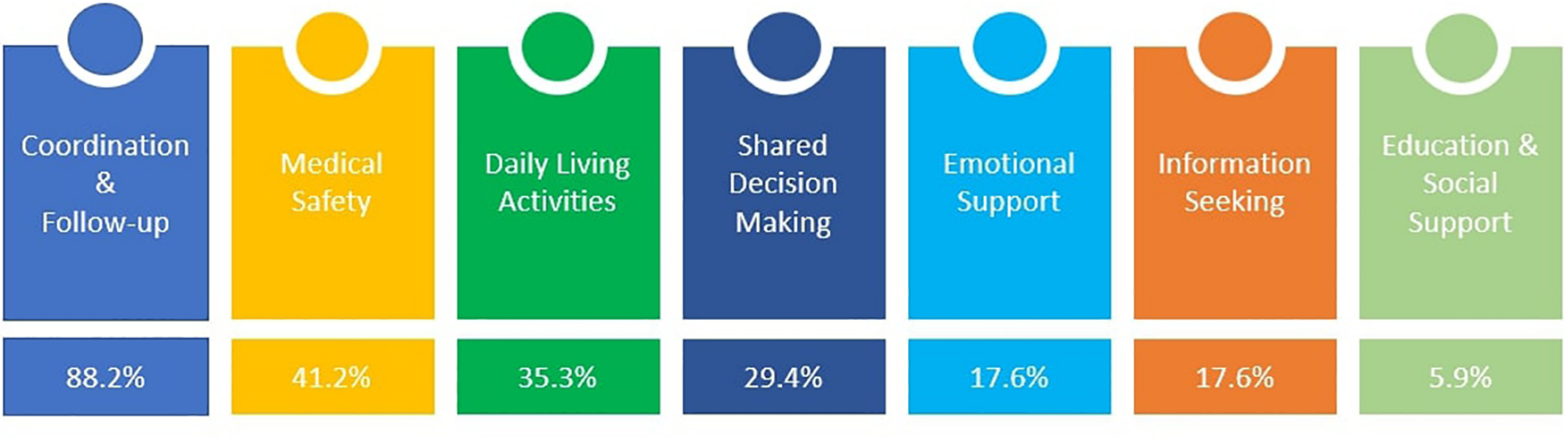
The needs are addressed by technology designed for CMC and their caregivers.

Figure 5 shows the needs addressed by the solutions of the selected studies. Most of the studies addressed the coordination and follow-up issue (88.2%, *N* = 15/17). A total of (*N* = 7) studies addressed the need for medical safety, (*N* = 6) studies addressed the daily living activities, and (*N* = 5) studies addressed shared decision-making issues. Only three studies raised the need for information seeking and emotional support, and one study explored the need for education and social support.

**MQ7 & MQ8: Who are the target audience in CMC consumer technology studies? Who are the users that literature tried to support most?**

All the studies supported the caregivers (100%, *N* = 17), and 3 of them involved the doctors responsible for them. The studies targeted CMC and their caregivers (parents or professional caregivers), doctors, designers of technology, policymakers, hospital managers, and all the stakeholders involved in the care of the CMC.

**MQ9: What is the type of consumer informatics used?**

The selected studies covered various types of consumer informatics. As shown in (Figure 3), most studies used telehealth or telemedicine (*N* = 8/17). Mobile health was the second most used technology (*N* = 5/17), followed by the online portals, where three included studies explored its impact on CMC and their caregivers.

## Discussion

According to our findings, interventions to address the demands of care experienced by CMC families are emerging and promising. There is an increasing interest in consumer informatics that aim to support CMC and their caregivers in the past decade. This can be explained by the fact that more attention is given to using health information technologies in CMC home care. In recent years, there has been emphasis on capturing health information electronically and modernizing health communication flows (34). Improvements in medical technologies have led to advanced opportunities for home care and increased survival rates among this population (2). Most of these efforts are centered around traditional clinical settings and are driven by providers. However, many health and care activities occur outside of clinical settings and are not systematically documented or integrated into the clinical system. As a result, little information is captured for each patient, which can adversely affect clinical decision-making. Children who have special needs, like CMC, face more significant challenges in this regard (34).

Positive findings of caregivers' and patients' experiences support were noted and mirrored a broader body of established health literature for other populations (35, 36). Interventions identified in this review sought to directly target CMC and their caregivers' needs by providing consumer technology-based interventions to care for children at home, emphasizing collaboration between families and healthcare providers.

### Coordination and follow up, information exchange, and shared decision making

Effective care coordination is a critical strategy for improving quality and safety in CMC care. Prior studies have shown that event notifications help providers learn about the patient's background and prompt, timely interventions when needed, whether medical or related to care coordination and referral (37). Looking closely at impact, most of the studies in this review focused on the need for coordination and follow-up. Wang et al. reported that a model used through the patients' portals helped caregivers be active participants in the care of the CMC by sharing care coordination responsibilities with the “core team” composed of clinicians and care coordinators (33). The model shares reminders and notifications for follow-up and allows caregivers to enter information about the child's health situation to facilitate the team's access to information. It also allows them to be integral partners in the care team, resulting in a system in which “care is happening with them, not to them” (33). The caregivers' need to receive reminders and track health data was also shown by Cheng et al. suggesting mobile health as a solution for coordination (32). Telehealth was also shown to have a practical impact on care coordination. It addressed the caregivers' unmet needs for care coordination (21). In addition, good home monitoring impacts CMC health outcomes as it can help manage health complications, prevent emergency readmissions, and reduce unnecessary unplanned visits. Mobile technology allows caregivers to track early symptoms that commonly precede acute escalations of their child's conditions (23). Telehealth is also effective in reducing unplanned visits over time (19).

### Emotional support and education & social support

When caring for children with chronic medical needs, parents and family members are subjected to extraordinary stress (8). Stress may arise from substantive emotional, psychological, social, and financial issues associated with the caregiving role and stress from marital and family obligations (38). As CMC is highly reliant on family caretakers, these additional stresses may adversely affect the parent-child relationship and contribute to the caregiver's poor health (39). Studies developed interventions to support caregivers' emotional well-being (8). Our review found that consumer technologies can contribute to managing this stress. For example, caregivers' uncertainty can be controlled by teaching them how to deal with unusual situations and giving them more flexibility in the complex tasks they are dealing with through mobile applications (32). Educating caregivers can help them gain self-esteem and trust in their capabilities in caring for their children, reducing their anxiety levels (32).

### Daily living activities and medical safety

CMC patients might experience medication errors due to the complexity of their care and their inherent fragility both in the hospital and home environment. Fragmented patient care and miscommunication are a source of errors for CMC, who transition between healthcare settings and practitioners (40). Injuries often occur in CMC care due to parents failing to fill prescriptions or poor communication of dose changes (41). In the pediatric arena, providers and caregivers must provide care to CMC without specific training or guidelines to support them (41). This may pose a threat to the child's safety. Thus, more control over daily activities is necessary to ensure this safety. Besides, there is no comprehensive support available to efficiently support the management or sharing of information through electronic health records, which means health care providers and families need to exert considerable effort to achieve this on their own (42).

While perhaps not accessible by all families, consumer informatics represents an opportunity for caregivers to ensure their children's medical safety in their day-to-day activities. Individualized services offered through text messaging options to caregivers can help build their confidence to request information about what they should do to ensure their children's safety (22). This can help prevent errors and provide better medical safety in CMC care. Telehealth can also help facilitate a safe and effective transition of CMC care from hospitals to homes (27, 29).

### Challenges of consumer informatics' adoption

The literature shows that consumer informatics tool provides promising opportunities to support CMC family caregivers. Although feasibility studies show caregivers' high acceptability of these technologies, it is noteworthy that these participants also raised several concerns. It remains essential to address these issues before implementing more technologies to support home monitoring and CMC caregiving to ensure effective acceptance of consumer technologies. First, consumer informatics is not accessible by all caregivers (32). It is critical to explore factors leading to CMC caregivers' lack of access to consumer informatics tools, and enable equitable access, especially for CMC parents from underserved populations. If financial factors hinder access, local clinics, agencies, or insurance providers can explore the feasibility of offering free access to such technologies.

It is also important to use some well-validated methods such as the technology acceptance model to better understand factors that influence the actual use of these consumer informatics by CMC caregivers. There should also be more studies to explore the effectiveness of consumer technologies in CMC care. There should be more objective studies beyond only capturing end users’ perceptions to better understand the direct impact of consumer technologies on CMC care management and outcomes. Finally, technology may facilitate some tasks for caregivers; it is noteworthy that it may add more burden to the providers and more workload to what they already have. Some providers complained about the extra burden that the consumer informatics added to their workload and the trustworthiness of the information shared by the non-healthcare providers (33).

### Future research & limitations

We believe that our study will provide researchers and practitioners with relevant information regarding the current needs of CMC family caregivers and how these needs are addressed using consumer informatics solutions and recommendations for future publications. For future work, we intend to develop a conceptual framework that can help evaluate the technologies designed by highlighting the design problems and the usability of the tools to offer sustainable CMC consumer informatics solutions. However, this study also has some limitations that are worth acknowledging. For example, we only included three databases PubMed, Web of Science, IEEE Xplore, but did not consider others like Scopus. It should be noted that a recent study showed that slight differences exist between the scientific literature covered in Scopus and Web Of Science, which will result in a large number of duplicates in reviews that include both (43). In addition, we added the term “special needs” to the list of Mesh terms used in the search to ensure we are not missing any study dealing with CMC. This resulted in many articles being out of scope, which was time-consuming in the filtering process. It is important to note that both authors have experience conducting systematic literature reviews (44).

## Conclusion

This paper conducted a systematic mapping literature review, resulting in 17 final publications providing an overview of the available literature on CMC consumer informatics. Consumer informatics tools have the potential to support the CMC family caregivers' needs in information seeking, shared decision-making, care coordination, and ensuring medication safety and education. Practitioners, policymakers, and technology designers should further explore tool-based proof of technology effectiveness that addresses caregivers' needs, which can help with overall CMC home care. This study provides researchers and practitioners with relevant information regarding the current CMC needs, how they are addressed with consumer informatics solutions, and recommendations for future publications. For future work, we intend to develop a conceptual framework that can help evaluate the usability and effectiveness of Consumer informatics tools tailored to the specific needs of CMC patients and their family caregivers.

## Summary table

**Table:**

* CMC consumer informatics is a promising research field to present novel initiatives and approaches to assist in managing the caregivers’ workload
* Telehealth and telemedicine was the most used type of consumer informatics for children with medical complexity (CMC) support, followed by mobile health.
* Consumer informatics help CMC by addressing seven needs: Coordination & follow-up, medical safety, education & social support, daily living activities, shared decision making, information seeking, and emotional support.
* Most of the efforts of consumer informatics for CMC and their caregivers support focused on ensuring good coordination and follow-up for them.
